# H_1_-antihistamines are associated with lower prevalence of radiographic knee osteoarthritis: a cross-sectional analysis of the Osteoarthritis Initiative data

**DOI:** 10.1186/s13075-018-1619-7

**Published:** 2018-06-07

**Authors:** Ivan Shirinsky, Valery Shirinsky

**Affiliations:** Laboratory of Clinical Immunopharmacology, Federal State Budgetary Scientific Institution, Research Institute of Fundamental and Clinical Immunology, 6 Zalesskogo Street, 630047 Novosibirsk, Russia

**Keywords:** Osteoarthritis, Knee osteoarthritis, Outcomes research

## Abstract

**Background:**

There is growing evidence that mast cells (MCs) play a role in knee osteoarthritis (OA). H_1_-antihistamines block H_1_-receptors of histamine, which is an important mediator of MCs. There is a lack of data on whether H_1_-antihistamines can influence OA. We hypothesized that the use of H_1_-antihistamines may be linked to the reduced prevalence of knee OA.

**Methods:**

Baseline data from the Osteoarthritis Initiative cohort were analysed cross-sectionally. Unadjusted and adjusted logistic regression models were performed to compare the prevalence of knee OA in H_1_-antihistamine users and non-users. Generalized estimating equations were used to adjust for the correlation between knees. Knee OA was defined as (1) Kellgren-Lawrence (KL) grade ≥ 2 or total joint replacement or (2) KL grade ≥ 2 and joint space narrowing or total joint replacement.

**Results:**

The analysed sample consisted of 8545 knees (664 knees of H_1_-antihistamine users and 7881 knees of H_1_-antihistamine non-users). The use of H_1_-antihistamines was associated with reduced prevalence of knee OA in unadjusted and adjusted models using both the first (adjusted OR, 0.77; 95% CI, 0.62, 0.96; *P* < 0.02) and second (adjusted OR, 0.75; 95% CI, 0.62, 0.93; *P* < 0.008) definitions of knee OA.

**Conclusions:**

H_1_-antihistamines are associated with a reduced prevalence of knee OA. The findings indicate that this class of drugs should be further evaluated for possible structure-modifying properties in knee OA.

## Background

Mast cells (MCs) have long been considered to be inflammatory cells involved primarily in parasitic infections and allergic reactions. In recent years, an emergent role has been described for MCs in various chronic inflammatory diseases, including cancers [1], rheumatoid arthritis [2], atherosclerosis, obesity and diabetes [3]. In osteoarthritis (OA), MCs are prevalent in synovial tissue, and their presence is associated with structural damage [4]. The primary action of histamine H_1_-receptor blockers (H_1_-antihistamines) is to block the effects of histamine on its specific receptors. In addition, many H_1_-antihistamines have anti-inflammatory effects and are able to stabilize the membranes of MC, leading to decreased release of multiple mediators of MC [5].

Therefore, H_1_-antihistamines might be a candidate class of drugs to prevent and treat OA. Currently, there is a lack of data on the effects of H_1_-antihistamines in knee OA in humans. In one exploratory study, H_1_-antihistamines were linked to decreased progression and less pain in knee OA [6].

We hypothesized that H_1_-antihistamine use may be linked to reduced prevalence of knee OA. To test this hypothesis, we evaluated the cross-sectional association between the use of oral H_1_-antihistamines and radiographic knee OA using the data from the publicly available Osteoarthritis Initiative (OAI) cohort.

## Methods

In these cross-sectional analyses we compared the knees of OAI participants taking H_1_-antihistamines at baseline with the knees of control participants. For the present study, we used longitudinal data obtained from the OAI, which is publicly available at http://oai.epi-ucsf.org. Specific datasets used were “MIF00” (version 0.2.2), “AllClinical00” (version 0.2.2), “Enrollees00 (version 17)”, “kXR_SQ_BU00” (version 0.6), and “Outcomes99” (version 8). The detailed information about the OAI protocol can be found elsewhere [7]. The OAI cohort consists of a progression subcohort (patients with symptomatic tibiofemoral knee OA, *n* = 1390), an incidence subcohort (subjects with increased risk of OA, *n* = 3284) and a reference control subcohort (*n* = 122). In this analysis we used the cross-sectional baseline data from both the progression and incidence subcohorts. The main inclusion criteria were the following: age between 45 and 79 years for both subcohorts, symptomatic tibiofemoral knee OA for the progression subcohort, and the presence of established or putative risk factors for incident knee OA for the incidence subcohort. The OAI subjects were recruited and enrolled between February 2004 and May 2006 at four recruitment centres in the United States. This study received ethical approval from each recruitment centre. All participants provided written informed consent. The prespecified sample size was 5000 women and men (4000 in the incidence subcohort, 800 in the progression subcohort). The sample size was expected to provide an adequate number of knees with incident and worsening OA-related structural and clinical changes to achieve the primary aims of the OAI study.

### Clinical measures

Height was measured in millimetres using a calibrated, wall-mounted stadiometer. The measurement was performed twice with the subject in light clothing, without shoes, and during inspiration. Body weight was measured in kilograms with a calibrated, standard balance beam scale. The measurement was performed twice with the subject in light clothing without shoes, heavy jewellery or wallets. Body mass index (BMI) was calculated based on weight (in kg) divided by height (in cm) squared. Smoking history and education status were assessed using self-administered questionnaires. Prior knee surgery, family history of knee replacement, and Physical Activity Scale for the Elderly (PASE) were evaluated using interview.

To acquire information about the use of H_1_-antihistamines, a medication inventory method was used wherein the participants brought in all of the medications they were currently taking, and the brand name, generic name or active ingredients were recorded and matched to an entry in an online medication dictionary [8]. Only participants who reported taking H_1_-antihistamines for more than 1 year prior to baseline were included in the analyses.

### Radiographic assessment

Posteroanterior weight-bearing knee radiographs were performed annually using a Synaflexer frame (Synarc, San Francisco, CA, USA), which allowed a fixed, standardized and reproducible knee position. X-ray interpretation was performed centrally at Boston University by three readers. In case of a disagreement about the presence of radiographic OA, the reading was adjudicated by a panel of three readers. A consensus reading was achieved when at least two of the three readers agreed.

The initial incidence and progression OAI subcohort assignments were based primarily on Kellgren-Lawrence (KL) readings at the OAI clinical centres. Because further central assessments could provide different grading results, assignment to incidence and progression subcohorts might not reflect a participant’s knee OA status at baseline.

In the OAI, there were two definitions of knee OA based on central reading: (1) KL grade ≥ 2 or total joint replacement and (2) KL grade ≥ 2 and joint space narrowing (JSN) or total joint replacement. We used both definitions as outcomes of our study.

### Statistical analysis

Continuous variables are presented as mean (SD), and categorical variables as number (percent). Although most of the continuous variables were not normally distributed, we used mean (SD) as the preferred statistic even in the setting of non-normally distributed data [9]. Unadjusted and adjusted logistic regression models were used to assess the association between the use of H_1_-antihistamines and radiographic knee OA. Generalized estimating equations (GEEs) were used to adjust for the correlation between knees. GEE allows for not using imputation methods for the missing data, because the participants with missing data are not excluded from the analysis [10]. The models were adjusted for BMI, age, race, sex, smoking history, education status, history of prior knee surgery, family history of knee replacement, PASE and subcohort assignment. These potential confounders were selected on the basis of literature and clinical plausibility. The adjustment for the subcohort assignment was carried out because the inclusion criteria for both subcohorts were different.

#### Sensitivity analyses

We performed sensitivity analyses using the definition of knee OA as a KL score ≥ 2 (not including knee replacement in the definition).

## Results

**Fig. 1:**
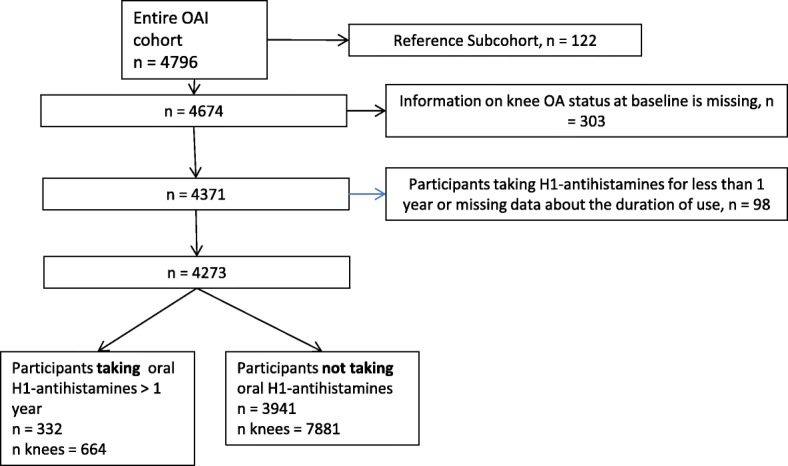
Flowchart of participants selected for the analyses

A sample of 4273 OAI participants (8545 knees) was analysed, and 332 of them (664 knees) used histamine H_1_-receptor blockers at baseline (Fig. 1). The vast majority of H_1_-antihistamine users were taking second-generation H_1_-antihistamines. The compared groups consisted of middle-aged, overweight participants. There were 1518 (25%) knees with radiographic OA in the incidence subcohort and 1537 (61.4%) knees with radiographic OA in the progression subcohort using the first definition of knee OA. According to the second definition of knee OA, there were 2028 (33.6%) and 1834 (73.3%) knees with radiographic OA in the incidence and progression subcohorts, respectively.

In relative terms, the prevalence of radiographic knee OA was 15.5% and 10.8% lower in the H_1_-antihistamine users group using the first and second definitions of radiographic knee OA, respectively. This difference was due to a lower proportion of patients with bilateral knee OA in the H_1_-antihistamine users group (26.5% and 17% less patients with bilateral knee OA using first and second definitions of knee OA, respectively) (Table 1).

**Table 1:** Characteristics of participants taking and not taking histamine H_1_-receptor antagonists

	Participants taking H_1_-antihistamines	Participants not taking H_1_- antihistamines
No. of participants	332	3941
No. of knees	664	7881
Age, yr	59.84 (8.17)	61.45 (9.23)
Sex		
Female	94 (28.3)	1712 (43.4)
Male	238 (71.7)	2229 (56.6)
BMI, kg/m^2^	29.18 (4.86)	28.64 (4.79)
Race		
Other non-white	3 (0.9)	64 (1.6)
White or Caucasian	261 (78.6)	3156 (80.1)
Black or African American	64 (19.3)	685 (17.4)
Asian	4 (1.2)	32 (0.8)
History of knee surgery	73 (22.0)	936 (23.8)
Family history of knee OA	60 (18.1)	611 (15.5)
PASE	160.38 (82.74)	161.68 (82.23)
Education		
Less than high school graduate	6 (1.8)	136 (3.5)
High school graduate	38 (11.4)	489 (12.4)
Some college	79 (23.8)	939 (23.8)
College graduate	63 (19.0)	846 (21.5)
Some graduate school	28 (8.4)	319 (8.1)
Graduate degree	116 (34.9)	1183 (30.0)
Smoking history		
Never	180 (54.2)	2056 (52.2)
Current	19 (5.7)	242 (6.1)
Former	129 (38.9)	1585 (40.2)
Current but never regular	2 (0.6)	6 (0.2)
WOMAC subscales		
WOMAC pain, right knee	2.93 (3.45)	2.42 (3.12)
WOMAC pain, left knee	2.70 (3.65)	2.28 (3.33)
WOMAC function, right knee	9.75 (11.28)	7.75 (10.19)
WOMAC function, left knee	9.92 (12.52)	7.92 (11.02)
KL, knees (%)		
0	256 (38.6)	2904 (36.8)
1	138 (20.8)	1386 (17.6)
2	176 (26.5)	2129 (27.0)
3	71 (10.7)	1135 (14.4)
4	18 (2.7)	272 (3.5)
Patients with knee OA, defined as KL ≥ 2 with JSN, or knee replacement (%)	145 (43.67)	1913 (48.54)
Unilateral, no. of patients (%)	87 (26.2)	974 (24.7)
Bilateral, no. of patients (%)	58 (17.5)	939 (23.8)
Patients with knee OA, defined as KL ≥ 2, or knee replacement (%)	181 (54.52)	2318 (58.84)
Unilateral, patients (%)	92 (27.7)	1044 (26.5)
Bilateral, patients (%)	89 (26.8)	1274 (32.3)
JSN, medial compartment, knees (%)		
0	441 (66.4)	4985 (63.3)
1	154 (23.2)	1734 (22.0)
2	58 (8.7)	912 (11.6)
3	6 (0.9)	195 (2.5)
JSN, lateral compartment, knees (%)		
0	608 (91.6)	7162 (90.9)
1	26 (3.9)	340 (4.3)
2	13 (2.0)	245 (3.1)
3	12 (1.8)	79 (1.0)
Knee replacement	5 (0.8)	53 (0.7)
H_1_-antihistamines		
Second-generation		
Fexofenadine	177 (53.31)	–
Cetirizine	85 (25.6)	–
Desloratadine	39 (11.75)	–
Loratadine	23 (6.93)	–
First-generation		
Diphenhydramine	7 (2.11)	–
Chlorpheniramine	4 (1.2)	–
Promethazine	5 (1.51)	–
Brompheniramine	1 (0.3)	–
Cyproheptadine	1 (0.3)	–
Dexbrompheniramine	1 (0.3)	–
Phenyltoloxamine	1 (0.3)	–
Pyrilamine	1 (0.3)	–
Duration of H_1_-antihistamine use		
1–3 yr	136 (40.96)	
3–5 yr	92 (27.71)	
More than 5 yr	104 (31.33)	
Study endpoints		
Radiographic knee OA, defined as KL ≥ 2 with JSN, or knee replacement, knees (%)	203 (30.6)	2852 (36.2)
Radiographic knee OA, defined as KL ≥ 2, or knee replacement, knees (%)	270 (40.7)	3592 (45.6)

*Abbreviations: OA* Osteoarthritis, *BMI* Body mass index, *KL* Kellgren-Lawrence grade, *PASE* Physical Activity Scale for the Elderly, *WOMAC* Western Ontario and McMaster Universities Osteoarthritis Index, *JSN* Joint space narrowing (grades 0–3)

Data are presented as the mean (SD) or number (%). Possible ranges for WOMAC pain score are 0–20. Possible ranges for WOMAC function score are 0–68

In the regression analyses, the use of H_1_-antihistamines was associated with lower prevalence of radiographic knee OA using both definitions in either crude or adjusted analyses (Table 2). The sensitivity analysis did not change the direction and significance of our results shown in Table 2.

**Table 2:** Association between the use of histamine H_1_-receptor antagonists and prevalence of radiographic knee OA

	Non-adjusted models			Adjusted models		
	OR	95% CI	*P* value	OR	95% CI	*P* value
Radiographic knee OA, defined as KL ≥ 2 with JSN	0.78	0.63 to 0.95	0.014	0.77	0.62 to 0.96	0.02
Radiographic knee OA, defined as KL ≥ 2	0.81	0.67 to 0.99	0.041	0.75	0.62 to 0.93	0.008

Note. The models were adjusted for BMI, race, age, gender, Physical Activity scale for the elderly (PASE), history of knee surgery, family history of knee OA, smoking status, education, and Subcohort assignment. OR – odds ratio, CI – confidence interval

## Discussion

In cross-sectional analyses of OAI data, H_1_-antihistamines were associated with decreased prevalence of radiographic knee OA. Our study had the following important limitations: a cross-sectional design and a lack of precise information about the duration and dose of antihistamine use. The presence of inclusion criteria may limit the generalizability of the results.

Our data are in line with an exploratory, hypothesis-generating study performed on longitudinal OAI data. In this study, antihistamine users, defined as those using antihistamines at the first four annual visits, showed a signal for reduced changes in joint space width during 36-month follow-up. The authors did not evaluate statistical significance and did not perform adjustment for possible confounders [6]. In contrast, our analysis was cross-sectional, we used a dichotomous outcome measure of radiographic knee OA, and our models were adjusted for multiple confounders. Thus, our data may be considered as an initial line of evidence that antihistamines may influence knee OA.

The demonstrated association between H_1_-antihistamine use and the decreased prevalence of knee OA may be explained in several ways. First, it is tempting to speculate that H_1_-antihistamines prevent knee OA by stabilizing the membranes of MC and blocking the effects of histamine, which is their major mediator.

The role of MCs in OA was suggested more than 20 years ago by several studies showing elevation of MCs and histamine levels in synovial fluids and synovial tissues from patients with OA [11, 12]. These findings were confirmed recently, and a trend towards an association between the number of MCs and increased KL grade was also found [4].

MCs are capable to produce a plethora of mediators which are released upon different stimuli via degranulation, secretion and exocytosis. Mediators stored in MC granules are represented by amines, proteoglycans, proteases, lysosomal enzymes, and cytokines [13]. Most of these compounds may be involved in the pathogenesis of OA. Thus, histamine induced an increase in the proliferation of human articular chondrocytes [14] and upregulated production of matrix metalloproteinase (MMP)-13 and MMP-3 by these cells via H_1_-receptors [15]. MC-derived polyamines, which are naturally occurring, positively charged polycations, are able to promote chondrocyte differentiation, which may result in OA [16]. MC-produced chymase has potent pro-inflammatory properties and plays a key role in MMP-9 and MMP-2 activation [17]. A recent meta-analysis showed a significant link between serum levels of MMP-9, MMP-2 and OA, suggesting a contribution of these MMPs to the pathogenesis of OA [18]. MCs have also been shown to synthesize, store and release nerve growth factor (NGF) [19], which is a promising new target for the treatment of pain in OA [20]. A recent study showed that NGF-induced production of prostaglandin D_2_ in joint MCs is critical for developing pain in OA [21]. One pilot observational study showed improvement of pain in patients with OA receiving therapy with anti-immunoglobulin E treatment, whose main effect is MC stabilization [22]. In addition, H_1_-antihistamines have been demonstrated to exert anti-inflammatory properties via down-regulation of nuclear factor-κB and suppression of secretion of various pro-inflammatory cytokines, including tumour necrosis factor-α, interleukin (IL)-6, IL-8 and granulocyte-macrophage colony-stimulating factor, by different cell types [23–25]. Thus, a growing body of data indicates that MCs and their mediators contribute to the pathogenesis of OA. Another way of suppressing MCs may be the use of peroxisome proliferator-activated receptor-α (PPARα) agonists [26]. Clinical improvement in erosive hand OA treated with the PPARα agonist fenofibrate in a small study [27] may in part be explained by the reduced activation of MCs.

Second, the findings may be due to inherent limitations of cross-sectional studies and the presence of unidentified confounders. There is a need of prospective studies evaluating the effects of H_1_-antihistamines on knee OA. Longitudinal observational studies may provide initial evidence that should be tested further in randomized controlled studies. It is agreed that to prevent several kinds of biases in the longitudinal analyses of observational cohort studies, one needs to employ a ‘new-user’ design that evaluates persons who were treatment-naïve at baseline and started the treatment of interest only after enrolment [28]. We were not able to perform this kind of analysis, because it would require a significantly larger sample size than that of the OAI dataset to gain sufficient statistical power using the selected knee OA outcomes. The strengths of this analysis are that it is based on a large population with a well-defined cohort, and we used standardised and reproducible procedures for knee radiograph acquisition, as well as an extensive adjudication process to determine KL and JSN grades.

## Conclusions

H_1_-antihistamines are associated with decreased prevalence of knee OA. In view of the absence of effective structure-modifying drugs for OA and the emerging role of MCs in OA, our findings provide an impetus for further studies evaluating H_1_-antihistamines in OA.

## Abbreviations

GEE: Generalized estimating equation
IL: Interleukin
JSN: Joint space narrowing
KL: Kellgren-Lawrence
MC: Mast cells
MMP: Matrix metalloproteinase
NGF: Nerve growth factor
OA: Osteoarthritis
OAI: Osteoarthritis Initiative
PASE: Physical Activity Scale for the Elderly
PPARα: Peroxisome proliferator-activated receptor-α
